# The application value of mean red blood cell volume and red blood cell volume distribution width combined with total serum bilirubin in the early screening of neonatal hemolytic disease

**DOI:** 10.1186/s12887-022-03812-2

**Published:** 2023-01-13

**Authors:** Hongxing Lin, Pingxiang Luo, Chen Liu, Xiaosong Lin, Chengwen Que, Wenhui Zhong

**Affiliations:** 1 Department of Blood Transfusion, Fujian Maternity and Child Health Hospital, No.18 Daoshan Road, Fuzhou, 350001 People’s Republic of China; 2 Department of Neonatology, Fujian Maternity and Child Health Hospital, No.18 Daoshan Road, Fuzhou, 350001 People’s Republic of China; 3 Clinical Laboratory, Fujian Maternity and Child Health Hospital, No.18 Daoshan Road, Fuzhou, 350001 People’s Republic of China

**Keywords:** Hemolytic disease of newborn, Mean corpuscular volume, Red blood cell volume distribution width, Total serum bilirubin

## Abstract

**Background:**

The hemolytic nature of hemolytic disease of the newborn (HDN) is described as the abnormal destruction and decomposition of red blood cells, causing heterogeneous manifestations such as abnormal red blood cell volume and morphology. Mean corpuscular volume (MCV) and red blood cell volume distribution width (RDW) are commonly used parameters related to red blood cell volume. Total serum bilirubin (TSB) is routinely monitored among newborns. This study aims to explore the value of MCV and RDW, combined with TSB, to improve the efficiency of HDN diagnosis.

**Methods:**

Three hundred eighty-eight children with HDN and 371 children with non-HDN pathological jaundice who were diagnosed and treated in the neonatal department of our hospital from January 2019 to December 2020 were included in the study. Clinical data collected include examination results of laboratory indicators, such as MCV, coefficient of variation of red blood cell volume distribution width (RDW-CV), standard deviation of red blood cell volume distribution width (RDW-SD), and TSB. The differences in the indicators between the two groups of children were retrospectively analyzed.

**Results:**

1) The detection rate of HDN in children in the early group was higher than that in the late group (*P* < 0.001). 2) The early-stage group had lower TSB levels and higher values of MCV, RDW-CV and RDW-SD (*P* < 0.001). Compared with the children in the non-HDN group, the indices in the HDN group were higher in the early stage (*P* < 0.001). 3) In the early stage, the TSB, MCV, RDW-CV, and RDW-SD were positively correlated with the diagnosis of HDN (*P* < 0.001). Early monitoring of TSB, MCV, RDW-CV and RDW-SD was valuable for HDN detection, the area under the curve (AUC) was 0.729, 0.637, 0.715, and 0.685, respectively (*P* < 0.001). 4) After a binary logistic analysis at TSB > 163.3 μmol/L, MCV > 96.35fL, and RDW-CV > 16.05%, the diagnosis rate of HDN increased (*P* < 0.001). The AUC of the HDN detected using the combined indicators was 0.841.

**Conclusion:**

At MCV > 96.35fL or RDW-CV > 16.05%, children with jaundice in three days of birth (especially children with TSB > 163.3 μmol/L) should be screened for HDN. A combination of TSB, MCV, and RDW-CV can improve the early detection rate of HDN, contribute to reduce the readmission rate and risk of hyperbilirubinemia.

## Introduction

Hemolytic disease of the newborn (HDN) is caused by the immunologic incompatibility of the blood type of the mother and child, which can result in anemia and jaundice in children. If it cannot be treated effectively in time, severe jaundice can be secondary to bilirubin encephalopathy, which can cause severe nervous system damage [1, 2]. As such, we must pay more attention to the early screening of HDN, which is very important for guiding the treatment plan and improving the prognosis of the patient. The hemolytic nature of HDN is described as the abnormal destruction and decomposition of red blood cells, causing heterogeneous manifestations such as abnormal red blood cell volume and morphology. The application of red blood cell morphology in the identification of hemolytic jaundice has been reported in the literature [3, 4]. Mean corpuscular volume (MCV) and red blood cell volume distribution width (RDW), the results of which are more objective and accurate than morphology, are commonly used parameters related to red blood cell volume. At present, the antibody screening of pregnant women is not routinely carried out. Total serum bilirubin (TSB) is routinely monitored among newborns in clinical practice. According to the changes in its level, three hemolysis tests are carried out to confirm HDN, but the tests take a long time; If the TSB level is low, HDN diagnosis may be missed. This study aims to explore the early changes of MCV and RDW in children with HDN, combined with bilirubin detection, to improve the efficiency of HDN diagnosis during hospitalization and reduce readmission rate.

## Materials and methods

### General information

#### Study subjects

The subjects of the study included 759 children. The treatment group consisted of 388 children with HDN who were treated in the neonatal department of our hospital from January 2019 to December 2020. The control group consisted of 371 children with non-HDN pathological jaundice who were treated in the same hospital during the same period. The following basic data of the children were collected: gender, gestational age, birth weight, hemoglobin, blood type and hemolytic workup due to red cell alloimmunization**.** Specifically, we analyzed the MCV, RDW, and TSB.

#### Criteria for inclusion in the study

①All full-term infants were born with a birth weight appropriate for their gestational age, and their weights were higher than 2500 g; ②the children had yellowish skin as the reason for medical treatment.

#### Criteria for exclusion from the study

①Patients with liver and biliary system diseases and incomplete clinical data were excluded. ②Other excluded factors: preterm birth, low birth weight. A total of 1053 subjects, 294 subjects were excluded.

### The diagnostic criteria for HDN

The diagnosis of HDN relies on the results of three hemolysis tests. Because of abnormal jaundice, children in both groups were sent for examination to determine its cause. Given the experimental results, including direct anti-human ball test (DAT) and RBC antibody identification (containing free antibody test and antibody release test), those with any two positive tests, or a single positive release test, can be diagnosed as HDN [5].

## Methods

### Groups of subjects

①Group according to experimental diagnosis: HDN group/non-HDN group; ②Group according to the peak period of domestic jaundice (4–6 days): early group (1–3 days)/late group (> 3 days). ③Children with Hb < 145 g/L were classified as the anemia group and those with Hb ≥ 145 g/L were classified as the non-anemia group [6].

### Method of determination

Referring to the “National Clinical Laboratory Procedures”[7], DAT adopted the test tube method and the free antibody experiment, antibody release experiment, and blood group identification were performed using the microcolumn gel method. The MCV, RDW, TSB, and other indicators were analyzed using fully automatic instruments.

### Instruments and reagents

The blood type test card and anti-human globulin card, as well as the matching WADiana automatic blood type tester, were provided by Beijing Banpers Technology and Trade Co., Ltd. Anti-human globulin antibody reagents were purchased from Shanghai Blood Biomedicine Co., Ltd. SSW type Microcomputer electric heating constant temperature water tank and KA-2200 SEROMATIC II centrifuge were also used. SysmexXE-5000 automatic blood analyzer was used to detect MCV and RDW, and an Abbott a16200 automatic biochemical analyzer was used to detect TSB.

### Statistical analysis

Statistical analysis of the data obtained was done in SPSS 22.0, and *P* < 0.05 indicated that the difference was statistically significant. 1) In the measurement data, those with normal distribution were represented by the mean ± standard deviation and differences were analyzed by Independent Sample T-test. Those with skewed distribution were represented by the median and the 25th-75th percentile, and the Mann–Whitney U test was performed on these data. 2) The chi-square test was used to compare the count data. 3) Spearman correlation analysis was used to test the correlation between MCV, RDW, TSB and confirmed HDN. 4) The receiver operating (ROC) curve was used to evaluate the value of MCV, RDW, and TSB during HDN screening. The area under the curve (AUC), sensitivity, and specificity were calculated, and the cutoff value was obtained. 5) The significance of the cutoff value used to screen the HDN was tested by performing univariate and multivariate binary logistic regression analyses.

## Results

### Clinical data analysis

The child’s sex, gestational age, birth weight, and results of the three hemolysis experiments submitted for inspection in the HDN group and non-HDN group are shown in Table 1. The detection rate of HDN in female children was higher than that in males (61%vs42.7%, *P* < 0.001). The gestational age of the HDN group was higher than that of the non-HDN group (*P* < 0.05). However, both groups of children were full-term children and their birth weight was greater than 2500 g, so it was considered that the gestational age had a negligible effect on the two groups of children. Furthermore, there was no difference in birth weight between the two groups (*P* > 0.05). The detection rate of HDN in the children in the early group was higher than that in the late group (63.4%vs32.8%, *P* < 0.001).

**Table 1:** Comparison of general information of HDN and non-HDN infants

	HDN group(*n* = 388)	Non-HDN group(*n* = 371)	χ^2^/t	*P*
Gender				
Male (n)	175	235	25.401	< 0.001
Female (n)	213	136		
Gestational age(W)	39.36 ± 1.05	39.16 ± 1.11	-2.605	0.014
Birth weight (g)	3329.85 ± 365.09	3288.25 ± 365.94	-1.567	0.117
Three experiments of hemolysis				
Early group (n)	288	166	68.585	< 0.001
Late group (n)	100	205		

*HDN* Hemolytic disease of the newborn

### Comparison of TSB, MCV, RDW-CV, and RDW-SD among the groups

All results are shown in Table 2. Compared with the late-stage group, the early-stage group had lower TSB levels and higher values of MCV, RDW-CV, and RDW-SD (*P* < 0.001). Compared with children in the non-HDN group, the indices in the HDN group were higher in the early stage (*P* < 0.001), while in the late stage, there was no difference in TSB and RDW-CV between the two groups (*p* = 0.504, 0.192, respectively). Furthermore, the HDN group had a higher MCV and RDW-SD than the non-HDN group (*P* < 0.001).

**Table 2:** Comparison of TSB(μmol/L), MCV(fL), RDW-CV(%) and RDW-SD(fL) among the groups

		Early group	Late group	Z	*P*
TSB	HDN group	230.7 (185.5, 284.6) ^a^	301.7(269.5, 341.9)^b^	-8.147	< 0.001
	Non-HDN group	152.4(60.4, 241.9)	295.4(261.8, 336.4)	-12.817	< 0.001
MCV	HDN group	99.6 (96.2, 102.6) ^a^	96.4(93.2, 98.4)^a^	-6.468	< 0.001
	Non-HDN group	96.3(93.2, 101.1)	93.8(91.1, 96.5)	-5.702	< 0.001
RDW-CV	HDN group	16.8 (16.0, 17.9) ^a^	15.2(14.7, 15.9)^c^	-10.528	< 0.001
	Non-HDN group	15.7(15.2, 16.7)	15.1(14.7, 15.7)	-6.871	< 0.001
RDW-SD	HDN group	59.1 (56.1, 64.0) ^a^	53.1(51.4, 56.1)^a^	-9.136	< 0.001
	Non-HDN group	55.2(52.2, 59.2)	51.8(49.9, 54.3)	-7.38	< 0.001

In the early stage, compared with the non-HDN group, ^a^ *P* < 0.001, Z values were -8.129, -4.88, -7.654, -6.557, respectively; In the late stage, compared with the non-HDN group, ^b^ *p* = 0.504, ^a^ *p* < 0.001, ^c^ *p* = 0.192 and Z values were -0.669, -4.425, -1.304, -3.563, respectively

*TSB* Total serum bilirubin, *MCV* Mean corpuscular volume, *RDW-CV* Coefficient of variation of red blood cell volume distribution width, *RDW-SD* Standard deviation of red blood cell volume distribution width

### Correlation between early TSB, MCV, RDW-CV, and RDW-SD and confirmed HDN

In the early stage, the indicators were positively correlated with confirmed HDN (R^2^ = 0.382, 0.229, 0.36, 0.309, respectively, *P* < 0.001), as shown in Table 3.

**Table 3:** Correlation between early TSB, MCV, RDW-CV and RDW-SD and confirmed HDN

Parameter	R^2^	*P*
TSB	0.382	< 0.001
MCV	0.229	< 0.001
RDW-CV	0.36	< 0.001
RDW-SD	0.309	< 0.001

*TSB* Total serum bilirubin, *MCV* Mean corpuscular volume, *RDW-CV* Coefficient of variation of red blood cell volume distribution width, *RDW-SD* Standard deviation of red blood cell volume distribution width

### ROC curve analysis

**Fig. 1:**
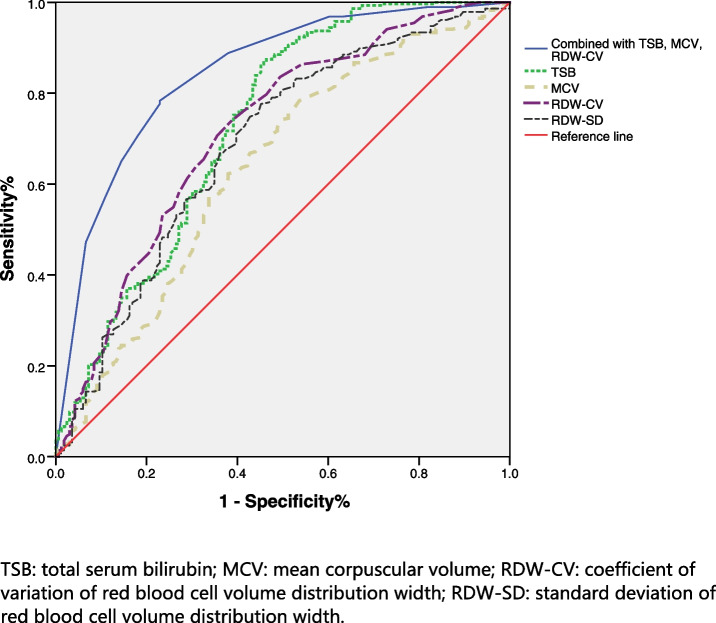
Analysis of ROC Curve of Early Diagnosis of HDN by Indicators

The cutoff values of HDN detected by early TSB, MCV, RDW-CV, and RDW-SD are shown in Table 4 and Fig. 1. All indicators were valuable for the diagnosis of HDN, and the AUC of each indicator was greater than 0.600 (*P* < 0.001).

**Table 4:** ROC curve analysis

Parameter	AUC	Cutoff	Sensitivity%	Specificity%	*P*
TSB	0.729	163.3	86.5%	45.2%	< 0.001
MCV	0.637	96.35	74.3%	49.4%	< 0.001
RDW-CV	0.715	16.05	74.1%	38.6%	< 0.001
RDW-SD	0.685	55.95	77.6%	45.2%	< 0.001

*TSB* Total serum bilirubin, *MCV* Mean corpuscular volume, *RDW-CV* Coefficient of variation of red blood cell volume distribution width, *RDW-SD* Standard deviation of red blood cell volume distribution width

### The effect of early monitoring of TSB, MCV, RDW-CV, and RDW-SD on the detection rate of HDN

After the binary logistic analysis at TSB > 163.3 μmol/L, MCV > 96.35fL, and RDW-CV > 16.05%, the diagnosis rate of HDN in children would increase (*P* < 0.001), as shown in Table 5. The AUC of the HDN detected using the combined indicators was 0.841, which was significantly higher than that of a single indicator, as shown in Fig. 1.

**Table 5:** The influence of early MCV, RDW-CV, RDW-SD, and TSB on the detection rate of HDN

Parameter		Univariable	*P*	Multivariable	*P*_adjust_
		OR(95% CI)		OR _adjust_(95% CI)	
TSB(μmol/L)	≤ 163.3^R^	7.747(4.913–12.215)	< 0.001	13.544(7.688–23.858)	< 0.001
	> 163.3				
MCV(fL)	≤ 96.35^R^	2.962(1.980–4.433)	< 0.001	2.904(1.538–5.483)	0.001
	> 96.35				
RDW-CV(%)	≤ 16.05^R^	4.526(3.008–6.811)	< 0.001	3.972(2.204–7.159)	< 0.001
	> 16.05				
RDW-SD(fL)	≤ 55.95^R^	4.247(2.810–6.418)	< 0.001	1.631(0.796–3.342)	0.182
	> 55.95				

OR values were adjusted for gender, TSB, MCV, RDW-CV, RDW-SD; R, references group

*TSB* Total serum bilirubin, *MCV* Mean corpuscular volume, *RDW-CV* Coefficient of variation of red blood cell volume distribution width, *RDW-SD* Standard deviation of red blood cell volume distribution width

### Significance of early MCV, RDW-CV, and RDW-SD in predicting anemia in children with jaundice

According to the cutoff value of each index for group comparison, when MCV or RDW-CV was greater than the critical value, the probability of anemia in children was higher (20.3%vs36.5%, 22.0%vs36.5%, respectively, *P* < 0.001). There was no difference in the RDW-SD grouping (25.8%vs33.4%, *P* = 0.095), as shown in Table 6.

**Table 6:** Significance of early MCV, RDW-CV, RDW-SD in predicting anemia

	MCV(fL)		RDW-CV(%)		RDW-SD(fL)	
	≤ 96.35	> 96.35	≤ 16.05	> 16.05	≤ 55.95	> 55.95
Anemia group (n)	32	108	39	101	40	100
Non-anemia group (n)	126	188	138	176	115	199
χ^2^	12.728		10.541		2.793	
P	< 0.001		< 0.001		0.095	

*MCV* Mean corpuscular volume, *RDW-CV* Coefficient of variation of red blood cell volume distribution width, *RDW-SD* Standard deviation of red blood cell volume distribution width

## Discussion

The bilirubin level of pathological jaundice is high and lasts for a long time. Therefore, it is very important to identify the cause of jaundice early and intervene in time to avoid severe hyperbilirubinemia. In our study, it can also be seen that the bilirubin level of the children gradually increased after birth. The late TSB of the children in the HDN and non-HDN groups was higher than that in the early stage, and there was no difference between the two groups in the late TSB. These findings indicate that as children age, high bilirubin levels are no longer conducive to identifying the cause of jaundice. However, the early stage HDN group had a higher TSB than the non-HDN group (230.7 vs. 152.4), indicating that the risk of hyperbilirubinemia in the HDN group was higher than that of the non-HDN group. Previous literature has also shown that HDN is a risk factor for neonatal hyperbilirubinemia [8]. In addition, the study found that the detection rate of HDN was higher than that of other causes of jaundice (63.4% vs. 32.8%) in the early stage, indicating that the HDN detection rate decreases with increasing age. Moreover, it was found that the incidence of HDN in females was higher than that of male children, which was consistent with the reported [9, 10].

This shows that early TSB monitoring in children with jaundice is not only beneficial for screening the possible cause but also for treatment. The current guidelines recommend measuring neonatal TSB within 24 h after birth, and carrying out follow-up laboratory tests based on changes in the TSB [11]. In China, the peak period of jaundice in term infants appears between the fourth and sixth day of life. With the improvement of medical standards, hospital stay among newborns has shortened. The peak period of jaundice is often after hospital discharge; however, there is a lack of follow-up examinations. Lack of awareness of the hazards of jaundice and insufficient attention to it, are important reasons for severe hyperbilirubinemia and even bilirubin encephalopathy [12, 13]. At present, there have been researches on the use of smartphone applications for bilirubin screening, supplementing TSB monitoring after discharge from the hospital [14]. This research focuses on the routine screening of TSB, combined with simple indicators, such as MCV and RDW, to improve the diagnostic efficiency of HDN during neonatal hospitalization (1–3 days of age), reducing the readmission rate and risk of hyperbilirubinemia.

Routine blood tests are one of the most basic blood tests for the monitoring and treatment of admitted children. A large number of blood cell-related parameters can be obtained, which is of great significance for the differential diagnosis of many diseases [15, 16]. Among these, MCV is a parameter that reflects the volume of peripheral RBCs. It has been reported that in ABO-HDN, the peripheral blood broken cell index and blood smear spherical red blood cell ratio are relatively high [17]. In this study, the children in the HDN group and the non-HDN group had MCV in the early stage greater than that in the late stage, and the MCV gradually decreased with the progression of the jaundice course, which was contrary to the development process of TSB. In contrast to TSB, the MCV of the HDN group was higher than that of the non-HDN group in both the early and late stages (99.6vs96.3, 96.4vs93.8, respectively). It was also found that the higher the early MCV, the higher the probability that the child was diagnosed with HDN (R^2^ = 0.229), and the correlation with HDN was higher than that of previously reported indicators (such as reticulocytes and lactate dehydrogenase) [18]. In particular, when the early MCV > 96.35fL

$$({OR}_{adjust}=2.904), HDN\ should\ be\ screened.$$

RDW is a parameter that reflects the volume heterogeneity of peripheral RBCs. It is automatically generated after the red blood cell volume is detected using a blood analyzer. Compared with observing red blood cells on a blood smear with the naked eye, it can more objectively reflect the degree of unequal size of red blood cells. Additionally, the instrument counts two parameters: RDW-CV and RDW-SD. In this study, the multivariate binary logistic analysis showed that early RDW-CV was an independent factor that improves the detection rate of HDN. Early-stage measurement of RDW-CV was greater than that of the late stage, and it decreased with the progression of the jaundice course, which is also opposite to the development course of the TSB. Similar to the TSB, there was no difference in the late detection value of RDW-CV between the HDN and non-HDN groups, but the early detection value was higher in the HDN group (16.8vs15.7). The study found that the higher the early RDW-CV, the higher the probability that the child will be diagnosed with HDN (R^2^ = 0.36), and the correlation with HDN was better than that of previously reported indicators [18], especially when the early RDW-CV > 16.05% (OR _adjust_ = 3.972), HDN should be screened.

MCV and RDW are traditionally used in the differential diagnosis and treatment of anemia. Recent studies have also found that MCV and RDW are related to the infection process [19–21], and RDW was found to be related to a variety of diseases [22–24], such as cardiovascular disease, diabetes, kidney disease, etc. In this study, the TSB, MCV and RDW-CV were independent factors that increased the early detection rate of HDN. The early measurement of MCV and RDW-CV was valuable for improving the detection rate of HDN. The AUC for each index was greater than 0.600. Combined with the TSB (with an AUC of 0.729) for screening HDN, the AUC was 0.841. Joint monitoring could increase the detection rate of early HDN compared with a single index. Although the specificity of each index was not high, the sensitivity was good, and the early value was increased. Contrary to the developmental process of TSB, they complement each other for observation. Therefore, MCV and RDW-CV can be used as early screening indicators, and combined with TSB, has application value in the early screening of the cause of jaundice.

In the study, it was also found that in the group with MCV > 96.35fL or RDW-CV > 16.05%, the probability of anemia in children was higher (20.3%vs36.5%, 22.0%vs36.5%, respectively). A few children underwent emergency blood transfusion to treat anemia symptoms, and then samples were taken for three hemolysis experiments. This will affect the results of the experiments due to the consumption of blood group antibodies, which may cause missed HDN detection. There are not many of such cases in this study, and there is still a lack of sufficient observation with regards to this. We can pay more attention to the dual role of MCV and RDW-CV in blood transfusion guidance and HDN screening in such children. Additionally, the reticulocyte count often rises in hemolytic conditions. Reticulocytes are larger than mature RBC, thereby elevating the MCV, and the mixture of reticulocytes with mature RBC elevates the RDW. Therefore, reticulocytosis should continue to be observed as the underlying mechanism for the observed MCV and RDW elevations. This study did not include cases diagnosed with suspected HDN and those with multiple causes of jaundice, such as HDN with premature delivery, infection, G6PD deficiency, etc. Thus, this study can continue to expand the data used to improve the comparison.

## Conclusion

In summary, MCV and RDW-CV can be used as early screening indicators, combined with TSB, to improve the early detection rate of HDN, and guide targeted treatment of HDN. This is as to be timely and effective in reducing the level of bilirubin, there by reducing the readmission rate and risk of hyperbilirubinemia.

## Data Availability

The datasets used and/or analyzed during the current study are available from the corresponding author on reasonable request.

## Abbreviations

HDN: Hemolytic disease of the newborn
MCV: Mean corpuscular volume
RDW: Red blood cell volume distribution width
RDW-CV: Coefficient of variation of red blood cell volume distribution width
RDW-SD: Standard deviation of red blood cell volume distribution width
TSB: Total serum bilirubin
